# Supplementary material for: Xanthium strumarium Fruit Extract Inhibits ATG4B and Diminishes the Proliferation and Metastatic Characteristics of Colorectal Cancer Cells
Source: Toxins (Basel). 2019 Jun 2;11(6):313. doi: 10.3390/toxins11060313 (PMC6628400; doi:10.3390/toxins11060313)
Supplement: Supplementary file 1 [file toxins-11-00313-s001.pdf]

Supplementary Materials: *Xanthium strumarium* Fruit Extract Inhibits ATG4B and Diminishes the Proliferation and Metastatic Characteristics of Colorectal Cancer Cells

Hsueh-Wei Chang, Pei-Feng Liu, Wei-Lun Tsai, Wan-Hsiang Hu, Yu-Chang Hu, Hsiu-Chen Yang, Wei-Yu Lin, Jing-Ru Weng and Chih-Wen Shu

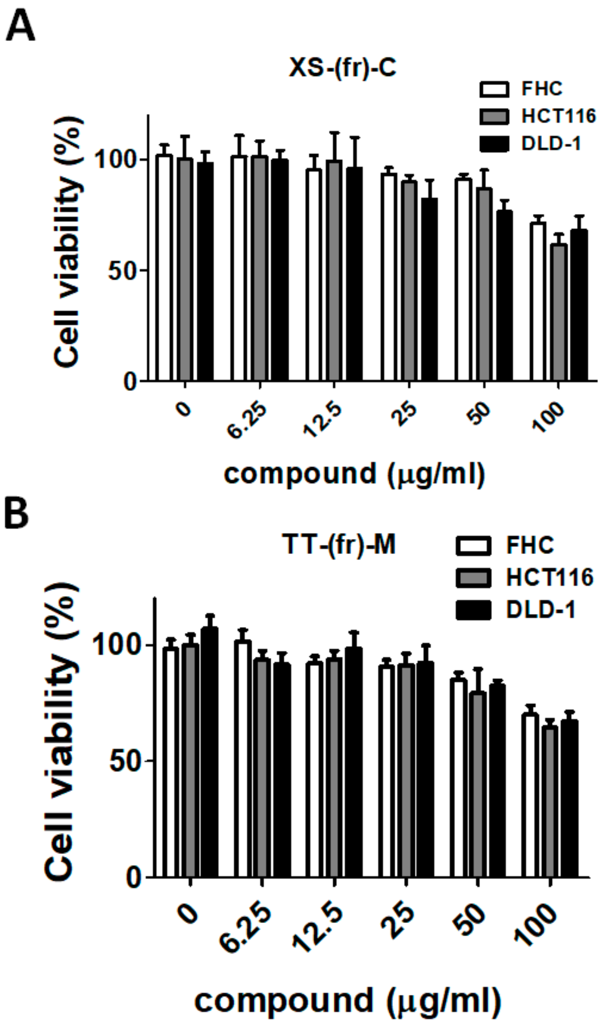

Figure S1. Effects of compounds on cell viability in colorectal cancer cells. Human colon normal epithelia cells FHC and colorectal cancer HCT116 and DLD-1 cells were cultured and treated with 2-fold diluted (100 µg/mL) extracts (A) XS-(fr)-C or (B) TT-(fr)-M in cultured medium (DMEM) for 24 h. The CellTiter-Glo reagent was added to cells, followed by measurement of the cellular ATP level to reflect cell viability.
